# Maternal antibiotic use and infections during pregnancy and offspring asthma: the Norwegian Mother, Father and Child Cohort Study and a nationwide register cohort

**DOI:** 10.1007/s10654-022-00897-y

**Published:** 2022-08-08

**Authors:** Aino K. Rantala, German Tapia, Maria C. Magnus, Lars C. Stene, Jouni J.K. Jaakkola, Ketil Størdal, Øystein Karlstad, Wenche Nystad

**Affiliations:** 1 Department of Chronic Diseases and Ageing, Norwegian Institute of Public Health, Oslo, Norway; 2 Center for Environmental and Respiratory Health Research, University of Oulu, Oulu, Finland; 3 Biocenter Oulu, University of Oulu, Oulu, Finland; 4 Centre for Fertility and Health, Norwegian Institute of Public Health, Oslo, Norway; 5 Clinic of Paediatric and Adolescent Medicine, Oslo University Hospital, Oslo, Norway; 6 Pediatric Research Institute, The Faculty of Medicine, University of Oslo, Oslo, Norway

**Keywords:** Asthma, Antibiotics, Infection, MoBa, Pregnancy, Childhood

## Abstract

Maternal antibiotic use during pregnancy has been linked to asthma risk in children, but the role of underlying infections remains unclear. We investigated the association of maternal antibiotic use and infections during pregnancy with offspring risk of asthma. We used two population-based cohorts: the Norwegian Mother, Father and Child Cohort Study (MoBa) (n = 53 417) and a register-based cohort (n = 417 548). Asthma was defined based on dispensed asthma medications at 7 and 13 years from the Norwegian Prescription Database. Self-reported information on antibiotic use and infections during pregnancy was available in MoBa, while registrations of dispensed prescriptions were used to classify use of antibiotics in the register-based cohort. Maternal antibiotic use during pregnancy was associated with asthma at 7 in both cohorts (adjusted risk ratio (aRR) 1.23, 95% CI 1.11–1.37 in MoBa and 1.21, 1.16–1.25 in the register cohort) and asthma at 13 in the register cohort (1.13, 1.03–1.23) after adjusting for maternal characteristics. In MoBa, the estimate was attenuated after adjusting for infections during pregnancy. Maternal lower and upper respiratory tract infections (aRR 1.30, 95% CI 1.07–1.57 and 1.19, 1.09–1.30, respectively) and urinary tract infections (1.26, 1.11–1.42) showed associations with asthma at 7. Register cohort also showed an increased risk of asthma in relation to maternal antibiotics before and after pregnancy. Our findings suggest that both maternal antibiotics and infections during pregnancy have a role in the risk of offspring asthma. However, results from the register cohort suggest that the effect of antibiotics may reflect the shared underlying susceptibility.

**Supplementary Information:**

The online version contains supplementary material available at 10.1007/s10654-022-00897-y.

## Introduction

Several studies provide evidence that maternal use of antibiotics during pregnancy is associated with increased risk of offspring asthma (Supplementary material Table S1) [1]. However, these studies were unable to evaluate a plausible role of underlying maternal infections during pregnancy, and could therefore not disentangle the effects of antibiotic use and infections. Studies have also reported associations between specific maternal infections related to antibiotic use such as respiratory tract infections (RTI) and urinary tract infections (UTI) during pregnancy and risk of childhood asthma [2–6]. However, findings are inconsistent, which might be due to the differences in type of maternal infection, control for confounding factors and assessment of childhood asthma [2].

The strongest associations reported between prenatal antibiotics exposure and asthma are for antibiotics prescribed for RTIs [7–9], which have led some authors to suggest that maternal use of antibiotics is a marker of maternal susceptibility to infections that is inherited by the child and increasing the risk of childhood asthma [8]. Consequently, it is necessary to investigate maternal infections during pregnancy as a potential confounder of the association between antibiotic use and asthma in offspring in detail with appropriate data.

Studies have evaluated the potential role of confounding in several ways. Maternal antibiotic use only before or after pregnancy was associated with an increased risk of asthma [8, 10, 11] and some studies indicate that the association was attenuated in sibling analyses [9, 12], supporting the conclusion that confounding might explain the association between antibiotic use during pregnancy and asthma. Findings for paternal antibiotic use as a negative control have been conflicting [10, 11, 13].

Earlier studies have not been able to address all these aspects of the relationship between prenatal antibiotic use and risk of asthma in the same study, and they have not been able to take into account infections during pregnancy and early childhood. The objective of this study was to investigate the association of maternal antibiotic use and infections during pregnancy with offspring risk of asthma. We addressed this research question using several methodological approaches in two study cohorts: the Norwegian Mother, Father and Child Cohort Study (MoBa) and a register-based cohort. First, we assessed the association between maternal antibiotic use and the risk of offspring asthma by taking into account the effect of infections during pregnancy in MoBa. Then we replicated previous studies without information on infections in a register-based cohort. Second, we assessed the association between maternal infections and the risk of offspring asthma in MoBa.

## Methods

### Study population

MoBa is a prospective population-based cohort that includes more than 114 000 children and their parents [14, 15]. Pregnant women were recruited at approximately 18 weeks of gestation between 1999 and 2008. The participation rate was 41%. All participants gave a written informed consent. We included all singleton children in MoBa, still alive and living in Norway, with information from the Medical Birth Registry of Norway (MBRN) and who had reached 8 years at the time of linkage to the Norwegian Prescription Database (NorPD) in July 2015. NorPD provided information on prescriptions of asthma medications. Information on infections and other potential confounding and mediating factors were from the MoBa questionnaires completed at 18 and 30 gestational weeks and when the child was 6 months and 18 months old. This left 53 417 children in the analysis (Supplementary material Fig. S1). The ongoing data collection in MoBa is approved by the Norwegian Health Registry Act.

The register-based cohort included all children born between 1 Januray 2004 (start of follow–up) and 31 December 2012, as registered in the MBRN (n = 541 036). Thus, the MoBa cohort is included in the register cohort. The NorPD provided information on dispensed prescriptions for antibiotics during pregnancy and early childhood, and prescription of asthma medications to mothers and children, but lacks information on infections. Only live-born singletons with a valid national identification number, who were alive and living in Norway at the time of data linkage, were eligible for the current study. We included children who had reached 8 years at the time of linkage to the NorPD in 31 December 2019 to investigate asthma at 7 years of age and children who had reached 14 years at the time of linkage to investigate asthma at 13 years. This left 417 548 (birth years 2004-11) and 67 098 (birth years 2004-05) children in the analysis when examining asthma at 7 and 13 years of age, respectively (Supplementary material Fig. S1). Norwegian legislation does not require consent from registered individuals to conduct health-related research using the national health registries.

This project was approved by the Regional Committee for Medical Research Ethics of South/East Norway.

### Exposure: antibiotics and infections

In MoBa, mothers reported infections and medication use during and after pregnancy in questionnaires completed at 18 and 30 gestational weeks and when the child was 6 and 18 months old [16]. The mother was asked to report any disease/infection of interest and medication used for these health problems. Antibiotics were grouped based on anatomical therapeutic chemical (ATC) pharmaceutical classification system (Supplementary material Table S2). Our analysis in MoBa included the total number of RTIs and UTIs. RTIs included upper respiratory tract infections (URTI) such as common cold, influenza, throat infection or sinusitis/ear infection and lower respiratory tract infections (LRTIs) such as pneumonia or acute bronchitis in mothers and additionally respiratory syncytial virus in the children.

In the register-based cohort, NorPD provided data on antibiotic prescriptions dispensed at Norwegian pharmacies. We considered maternal prescription of antibiotics 6 months before, during and 6 months after pregnancy as well as antibiotics prescribed for the child the first 18 months of life. We categorized antibiotics according to likely indication to be used as an indirect measure of RTIs and UTIs [17].

### Outcome: childhood asthma

The definition of asthma was based on dispensed asthma medications from the NorPD. Asthma at 7 years in both cohorts was defined as having at least two dispensed prescription for asthma medications: one in the 12 months preceding the 7th birthday, in addition to a second dispensed prescription within 12 months after the first. In the registry-cohort, we had also information on asthma at 13 years in children born 2004–2005. Asthma medications included inhaled beta(2)-agonists (ATC code R03AC), inhaled glucocorticoids (R03BA), combinations of inhaled beta(2)-agonists and glucocorticoids (R03AK), and leukotriene receptor antagonists (R03DC). In the register cohort, information on R03DC was not available.

### Other variables

For both cohorts available covariates included maternal characteristics such as age, parity, education, smoking during pregnancy, asthma and mode of delivery, and child characteristic such as sex, birthweight and gestational age. From MoBa additional variables included pre-pregnancy body mass index (BMI) and breastfeeding. Information on covariates was retrieved from questionnaires and MBRN.

### Statistical analysis

We assessed the associations of maternal antibiotics use and infections with offspring asthma using binomial regression, estimating risk ratios (RR) and 95% confidence intervals (CI). We fitted the exposures in the models as a categorical and as a binary variable (yes/no) as done also in the previous studies [8, 12].

First, we conducted multivariable analyses adjusting for all the potential confounding variables including maternal background characteristics (Table 1). In MoBa, we further mutually adjusted for the other exposures, e.g. when analysing maternal antibiotic use, we adjusted for maternal infections during pregnancy to evaluate confounding by indication. Furthermore, we adjusted models for child sex and potential mediators, such as gestational age, birth weight, breastfeeding and exposures in early childhood such as child antibiotics and infection by 18 months. Directed acyclic graphs (DAGs) in Supplementary Fig. S2 illustrate the conceptualization of our analysis and hypothetical causal model of (A) the association between maternal exposure to antibiotics during pregnancy and offspring asthma and (B) the association between maternal infections during pregnancy and offspring asthma. We were interested in potential direct effects and therefore, formal mediation analysis was not necessary.

We further conducted conditional analyses to distinguish the effect of antibiotics and infections during pregnancy in MoBa by analysing the association between antibiotics conditional on reporting a specific infection (indication) and offspring asthma.

We examined maternal use of antibiotics outside of pregnancy, i.e. 6 months before (only in register) and 6 months after pregnancy to evaluate confounding by underlying background characteristics influencing the likelihood of using antibiotics. In MoBa, we also used information on paternal antibiotic use as a negative control, which is recommended in observational studies to detect uncontrolled confounding [18]. In addition, we conducted a sibling-matched analysis using conditional logistic regression with maternal ID as grouping variable in the register-based cohort. Analyses were conducted in Stata version 16 (StataCorp, College Station, TX).

**Table 1:** Characteristics of the study populations

Characteristics	MoBa 2004- N (%)	Register cohort 2004-11 N (%)	Register cohort 2004-05, N (%)
**Total**	53 417 (100.0)	417 548 (100.0)	67 098 (100.0)
**Maternal age, years**			
< 25	5 301 (9.9)	70 537 (16.9)	11 042 (16.5)
25–29	18 097 (33.9)	130 472 (31.3)	21 121 (31.5)
30–34	21 003 (39.3)	138 157 (33.1)	23 194 (34.6)
>= 35	9 016 (16.9)	78 382 (18.8)	11 741 (17.5)
**Maternal parity**			
0	24 340 (45.6)	176 200 (42.2)	27 734 (41.3)
1	18 705 (35.0)	148 436 (35.6)	23 802 (35.5)
2	8 135 (15.2)	65 482 (15.7)	10 952 (16.3)
3 or more	2 237 (4.2)	27 430 (6.6)	4 610 (6.9)
**Maternal pre-pregnancy BMI**			
Underweight < 18.5	1 523 (2.9)		
Normal weight 18.5–24.9	34 212 (64.0)		
Overweight 25-29.9	11 582 (21.7)		
Obese > = 30	4 801 (9.0)		
missing	1 299 (2.4)		
**Maternal education**			
Less than high school/high school	19 475 (36.5)	188 530 (45.2)	31 520 (47.0)
College	33 745 (63.2)	212 368 (50.9)	33 439 (49.8)
Missing	197 (0.4)	16 650 (4.0)	2 139 (3.2)
**Maternal asthma during pregnancy**			
No	51 686 (96.8)	403 591 (96.7)	64 873 (96.7)
Yes	1 731 (3.2)	13 957 (3.3)	2 225 (3.3)
Maternal smoking during pregnancy*			
No	48 380 (90.6)	298 329 (71.5)	44 772 (66.7)
Yes, currently smoking	4 823 (9.0)	48 536 (11.6)	9 360 (14.0)
Missing	214 (0.4)	70 683 (16.9)	12 966 (19.3)
**Child sex**			
Male	27 326 (51.2)	214 315 (51.3)	34 288 (51.1)
Female	26 091 (48.8)	203 233 (48.7)	32 810 (48.9)
**Gestational age**			
>= 37 weeks	50 843 (95.2)	390 821 (93.6)	62 191 (92.7)
< 37 weeks	2 370 (4.4)	21 352 (5.1)	3 815 (5.7)
Missing	204 (0.4)	5 375 (1.3)	1 092 (1.6)
**Caesarean section**	7 153 (13.4)	66 224 (15.9)	10 513 (15.7)
**Birthweight (grams)**			
<2500	1 328 (2.5)	14 102 (3.4)	2 344 (3.5)
2500–3499	19 693 (36.9)	174 272 (41.7)	26 997 (40.2)
3500–4499	29 855 (55.9)	210 899 (50.5)	34 347 (51.2)
>= 4500	2 513 (4.7)	12 900 (3.1)	2 318 (3.5)
missing	28 (0.1)	5 375 (1.3)	1 092 (1.6)
**Breastfeeding**			
< 6 months	9 104 (17.0)		
6–12 months	23 710 (44.4)		
> 12 months	20 603 (38.6)		

*smoked after 18 weeks (MoBa) /still smoking end of pregnancy (register)

## Results

Characteristics of the study populations are shown in Table 1. In MoBa, the prevalence of asthma at 7 years was 4.1%. In the register-cohort, 3.8% of the children had asthma at 7 years, while 4.4% had asthma at 13 years of age. Overall, 15.6% of the children in MoBa were exposed to antibiotics during pregnancy, while 28.3% of children were exposed to antibiotics during pregnancy in the register-based cohort (Supplementary material Tables S4 and S6). Extended spectrum penicillins, such as amoxicillin were the most commonly used antibiotics in both study populations (Supplementary material Table S3).

### Maternal antibiotic use during pregnancy and offspring asthma

**Fig. 1:**
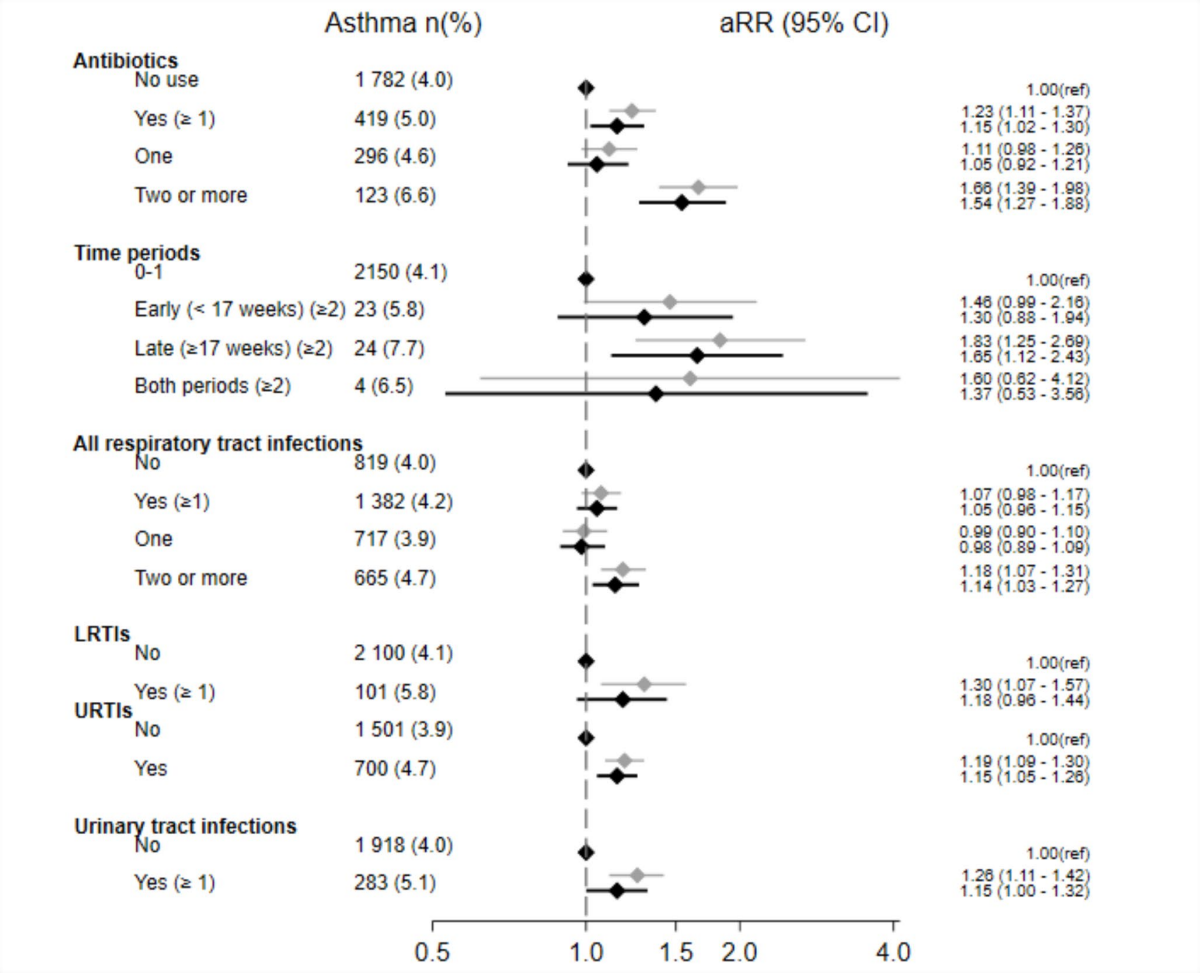
Forest plot of antibiotic use and infections during pregnancy and risk of asthma at 7 years in MoBa (n = 53 417). Risk ratios (aRR) adjusted for maternal characteristics such as maternal age, parity, pre-pregnancy BMI, asthma, smoking during pregnancy and education are in the top lines marked with a grey color. Risk ratios in addition adjusted for other exposures such as maternal respiratory and urinary tract infections during pregnancy (in the association between maternal antibiotics and asthma) or maternal antibiotic use (in the association between maternal infections and asthma) are in the bottom line, marked with a black color. LRTI, lower respiratory tract infections; URTI, upper respiratory tract infection

**Fig. 2:**
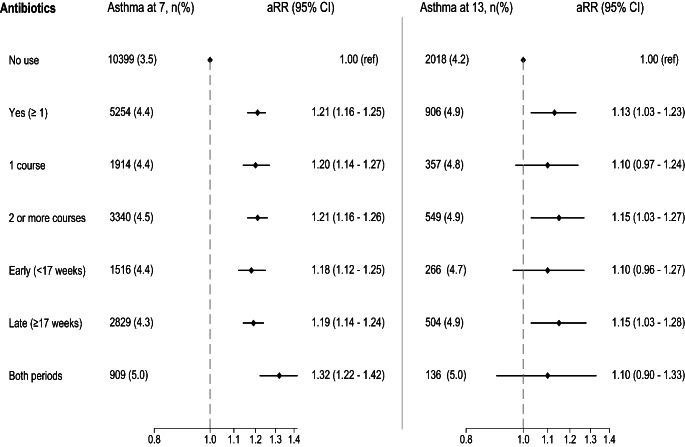
Forest plot of antibiotic use during pregnancy and risk of asthma at 7 (n = 417 548) and 13 years (n = 67 098) in the register-based cohort. Risk ratios (aRR) are adjusted for maternal characteristics such as maternal age, parity, asthma, smoking during pregnancy and education

Any maternal antibiotic use during pregnancy was associated with asthma at 7 year in both study populations (aRR 1.23, 95% CI 1.11–1.37 in MoBa and aRR 1.21, 95% CI 1.16–1.25 in register cohort) after adjusting for maternal characteristics (Figs. 1 and 2). In MoBa, the estimate decreased but remained significant after additional adjustment for RTIs and UTIs during pregnancy (aRR 1.15, 95% CI 1.02–1.30). We observed a dose-response pattern between antibiotics during pregnancy and offspring asthma in MoBa, where the use of antibiotics for two or more infections yielded an aRR of 1.54 (95% CI 1.27–1.88) after adjusting for infections. However, no dose-response association was observed in the register-based cohort.

In MoBa, exposure to antibiotics only in the late pregnancy (≥ 17 weeks) was associated with greater risk of asthma than use only in the early pregnancy (< 17 weeks), when comparing these mutually exclusive time windows to no use of antibiotics during pregnancy (Fig. 1). In the register-based cohort, antibiotic use during early and late pregnancy was similar (Fig. 2). After sibling-matched analysis of antibiotic use in the register cohort, associations disappeared (Supplementary material Table S8).

A weak association with any antibiotic prescriptions to mothers during pregnancy was also found with the risk of offspring asthma at 13 years (aRR 1.13, 95% CI 1.03–1.23) in the register-based cohort (Fig. 2). Similar to the association with asthma at 7 years, we did not observe dose-response pattern or time-dependent relationship in the association between antibiotic exposure during pregnancy and risk of asthma at 13 years.

Adjustment for child characteristics and exposures (mediators) further decreased the estimates (Supplementary material Table S4 for MoBa and Tables S6 and S7 for register-based cohort).

### Maternal infections during pregnancy and offspring asthma in MoBa

Children of mothers with any LRTIs (aRR 1.30, 95% CI 1.07–1.57), two or more URTIs (aRR 1.19, 95% CI 1.09–1.30), and any UTIs (aRR 1.26, 95% CI 1.11–1.42) during pregnancy, had an increased risk of asthma at 7 years (Fig. 1). URTIs and any UTIs showed consistent associations with child asthma after additional adjustment for antibiotic use during pregnancy (Fig. 1). Adjustment for offspring characteristics and exposures (potential mediators) slightly decreased the estimates (Supplementary material Table S5).

### Maternal antibiotic use conditional on specific infection and offspring asthma in MoBa

**Fig. 3:**
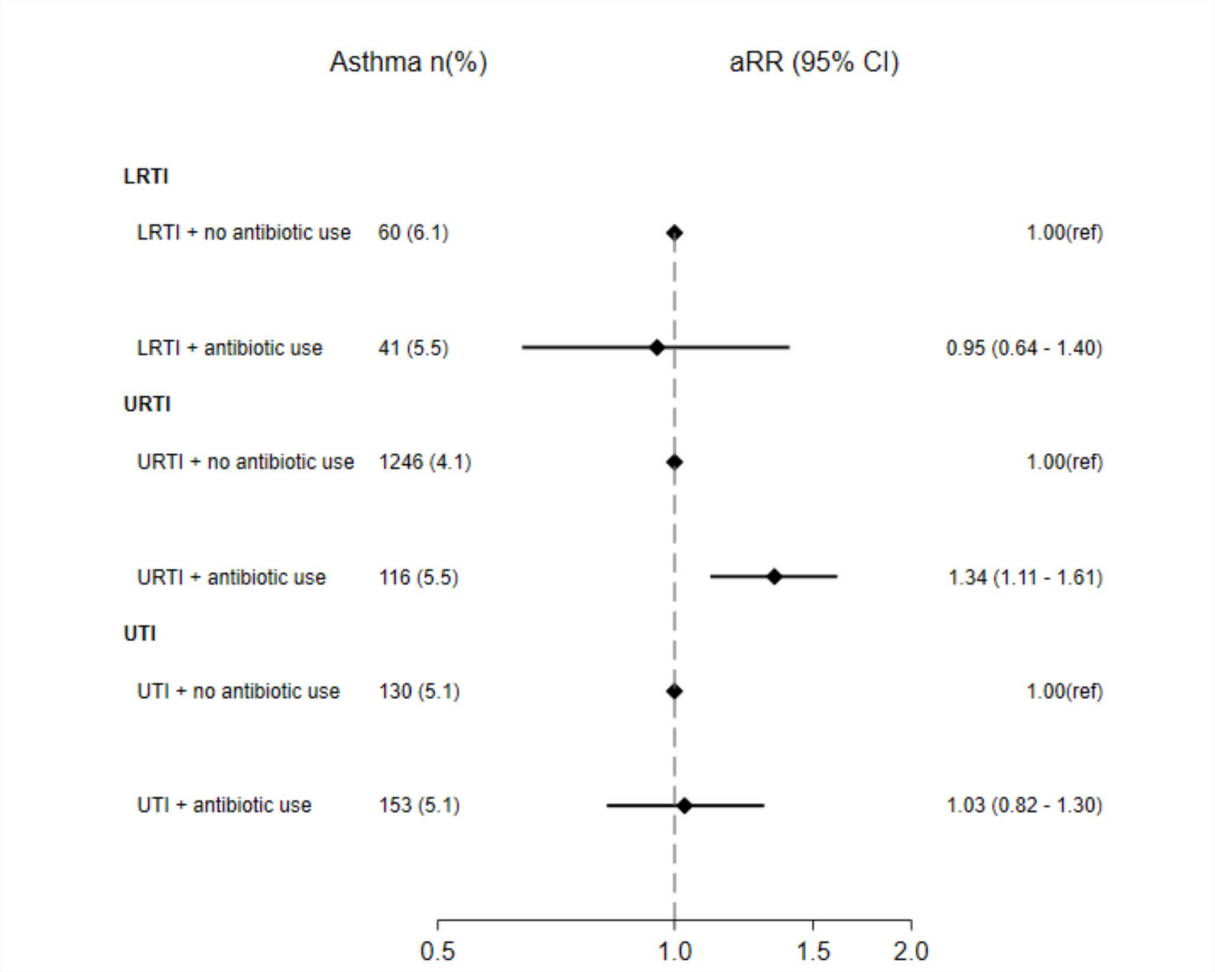
Forest plot of the conditional analyses where the risk of asthma at 7 years is compared between maternal LRTI (n = 1 732), URTI (n = 32 337) and UTI (n = 2 552) together with the use of antibiotics for that indication to indication without antibiotic use in MoBa. Risk ratios adjusted for confounders such as maternal age, parity, pre-pregnancy BMI, asthma, smoking during pregnancy and education

We observed that maternal antibiotic use during pregnancy was not associated with increased risk of offspring asthma beyond what was seen for those exposed to a particular indication such as LRTI and UTI (Fig. 3). However, there was a significantly higher risk associated with antibiotics use conditional on URTI. Supplementary material Table S9 shows that children of mothers with LRTI during pregnancy who did not use antibiotics had a higher risk of asthma (aRR 1.40, 95% CI 1.09–1.79) than children of mothers who used antibiotics for LRTI (aRR 1.26, 95% CI 0.93–1.71), when compared to children of mothers without any use of antibiotics or LRTIs. Similar result was observed related to UTIs during pregnancy (Supplementary material Table S9).

### Antibiotic use outside of pregnancy, paternal antibiotic use and offspring asthma

In the register-based cohort, maternal antibiotic use before and after pregnancy showed similar associations with the risk of asthma as use during pregnancy (Supplementary material Table S6 and S7). In MoBa, maternal use of antibiotics during pregnancy showed higher risk of child asthma than the use after pregnancy (Supplementary material Table S4), but information on antibiotic use before pregnancy was not available. Father’s use of antibiotics during pregnancy was not associated with offspring asthma at 7 years after adjustment for paternal characteristics (aRR 1.04, 95% CI 0.67–1.61) in MoBa.

### Prescription indication and offspring asthma in the register cohort

We observed increased risk of asthma at 7 years for antibiotics most likely prescribed for RTIs (aRR 1.18, 95% CI 1.13–1.24) and UTIs (aRR 1.16, 95% CI 1.11–1.22) after adjustment for confounders (Supplementary material Table S10). Association was observed also between prescription for RTIs (aRR 1.14, 95% CI 1.02–1.26) and asthma at 13 years.

## Discussion

In this population-based cohort study, both maternal antibiotic use and infections during pregnancy were associated with child asthma at 7 years. The associations between maternal antibiotic use during pregnancy and offspring risk of asthma decreased after adjusting for infections during pregnancy, which suggests that association is partly confounded by indication. Likewise, associations between maternal infections during pregnancy and offspring asthma was attenuated after accounting for use of antibiotics. However, different analytical approaches and cohorts result in somewhat diverging findings. Register cohort showed a positive association also between maternal antibiotic use outside of pregnancy and offspring asthma, and sibling-control analysis attenuated the main associations, which indicate that the observed effect of antibiotics might be confounded by factors shared by families.

### Strengths and limitations

Our study includes two large study populations, which both have their own strengths and limitations. MoBa is a large population-based prospective cohort that includes comprehensive questionnaire data collected during pregnancy and childhood. One significant advantage of our study is that we were able to consider a wide range of the maternal infections, which were not possible to take into account in our or other previous register-based studies.

We saw a lower prevalence of antibiotic use in MoBa than in the register-based cohort. MoBa includes information on self-reported use of antibiotics during pregnancy, whereas NorPD register includes information on dispensed prescriptions, which is likely to include loss of collection at the pharmacy to actual use of the drug leading to misclassification of exposure. It has been shown that half of wait-and-see prescriptions are never used [19] and therefore, information from the NorPD probably overestimates some of the actual use of antibiotics.

There is convincing evidence that dispensed asthma medication (yes vs. no) is an excellent indicator of the presence of asthma among Norwegian children [20]. The definition of asthma at 7 years of age, may include both allergic and non-allergic asthma [21], whereas asthma age 13 years may be more related to allergy [22]. Many conflicting findings on risk factors of asthma in previous studies might be a consequence of different phenotypic heterogeneities of asthma [7–9, 13, 23–25]. Our observation of modest associations of antibiotic exposure during pregnancy with asthma at 13 years is interesting, because earlier studies have reported that exposures like infections during early childhood might influence on asthma risk in young adulthood [26, 27], but effects of prenatal exposure are still unclear.

The participation rate in MoBa was 41%, which could introduce some selection bias. However, it has been shown that differences between the characteristics of MoBa study population and all Norwegian mothers giving birth in the same time period did not introduce any bias for studied perinatal exposure–outcome relationships [28]. We were able to minimize loss to follow-up selection bias by having a complete follow-up at 7 years. However, we had to exclude 36% of the children because of missing information from the follow-up questionnaires. The selection was primarily seen for according to maternal age, parity, and smoking, but prevalence of maternal and offspring asthma was similar.

### Synthesis with previous literature

Our findings are consistent with several other previous studies, but none of these studies were able to distinguish the effect of antibiotics from infections. Few studies have found stronger association for antibiotics likely given for RTIs interpreting that the association is confounded by indication [7–9]. Our findings support this interpretation, because maternal LRTIs during pregnancy were associated with childhood asthma when adjusted for maternal characteristics, but not when taking account childhood exposures including infections, which might indicate a mediating effect of the childhood exposures. Maternal antibiotic use might also be a marker for an increased propensity for antibiotic drug prescriptions in children and hypothesis are not necessarily exclusive [29].

We observed similar positive associations for both assumed indication categories (RTI and UTI) with asthma at 7 years in our register-based cohort. Likewise in MoBa using information on self-reported infections, we observed association for both RTIs and UTIs during pregnancy with asthma. Furthermore, maternal antibiotic use was not associated with increased risk of asthma beyond what was seen for those with LRTI or UTI during pregnancy suggesting that antibiotic use is not independently associated with childhood risk of asthma. However, it does not remove the overall association of antibiotics with offspring asthma, which we observed related to any antibiotic use and is also supported by the increased risk of asthma related to maternal antibiotic use conditional on URTI. To our knowledge, there are two previous studies reporting that maternal UTIs during pregnancy [3, 4] and two studies reporting that RTIs [5, 6] were associated with childhood asthma. Studies have suggested that findings might be explained by fetal programming of the developing immune system, as maternal infections are associated with a strong proinflammatory response that may predict the later development of allergic diseases [30].

Our results from the register-based cohort support the previous finding that observed increased risk of asthma in offspring exposed to antibiotics during pregnancy might reflect the shared underlying susceptibility. Similar to our register cohort, Stokholm et al. [8] and Örtqvist et al. [11] found that the association was similar before, during and after pregnancy, and sibling-control analysis has contributed to attenuation of the associations [7, 9, 12]. In contrast, antibiotic use after pregnancy was not associated with the risk of child asthma at 7 years in MoBa. In addition, paternal use of antibiotics showed no association with offspring asthma, which is in line with a study reporting no association between paternal antibiotics and asthma in children ≥ 2.5 years of age [11]. We also observed a dose-response pattern only in MoBa, but not in the register-cohort. Some of the previous register-based studies have found dose-response pattern from at least 3 courses upwards [10, 12]. We observed modest time-dependent association between antibiotics during early vs. late pregnancy in MoBa, like in other cohort studies [31, 32], but not in register cohort, which is consistent with other studies investigating trimester-specific associations in registers [10, 12, 33].

## Conclusions

Our study provides new information that both maternal antibiotic use and infections during pregnancy have a role in the risk of offspring asthma. However, different analytical approaches and cohorts result in somewhat diverging findings. Results from the register cohort support the previous finding that observed increased risk of asthma in offspring exposed to antibiotics during pregnancy is likely to reflect the shared underlying susceptibility.

## Electronic supplementary material

Below is the link to the electronic supplementary material.

Supplementary Material 1

## Data Availability

Data are available by contacting the Norwegian Mother, Father and Child Cohort Study administration (datatilgang@fhi.no).
