# Supplementary material for: Maternal antibiotic use and infections during pregnancy and offspring asthma: the Norwegian Mother, Father and Child Cohort Study and a nationwide register cohort
Source: Eur J Epidemiol. 2022 Aug 8;37(9):983–92. doi: 10.1007/s10654-022-00897-y (PMC9529693; doi:10.1007/s10654-022-00897-y)
Supplement: Supplementary file 1 — Supplementary Material 1 [file 10654_2022_897_MOESM1_ESM.docx]

Online-only Supplementary Material to:

**Maternal antibiotic use and infections during pregnancy and offspring asthma: the Norwegian Mother, Father and Child Cohort Study and a nationwide register cohort**

**Aino K. Rantala^1,2,3^, German Tapia^1^, Maria C. Magnus^4^, Lars C. Stene^1^, Jouni J.K. Jaakkola^2,3^, Ketil Størdal^1,5,6^, Øystein Karlstad^1^, Wenche Nystad^1^**

^1^Department of Chronic Diseases and Ageing, Norwegian Institute of Public Health, Oslo, Norway

^2^Center for Environmental and Respiratory Health Research, University of Oulu, Finland

^3^Biocenter Oulu, University of Oulu, Finland

^4^Centre for Fertility and Health, Norwegian Institute of Public Health, Oslo, Norway

^5^Clinic of Paediatric and Adolescent Medicine, Oslo University Hospital, Oslo, Norway

^6^Pediatric Research Institute, The Faculty of Medicine, University of Oslo, Norway

Corresponding author: Aino K. Rantala, Center for Environmental and Respiratory Health Research, University of Oulu, Finland. E-mail: aino.rantala@oulu.fi

**Supplementary Table S1. Summary table of previous studies on the association between any antibiotic use during pregnancy and childhood asthma.**

We used Ovid Medline (https://ovidsp.dc1.ovid.com) to search for the studies on the association between antibiotic use during pregnancy and childhood asthma with “antibiotics”, “pregnancy” and “asthma” as search terms. We selected studies, which investigated antibiotic use during pregnancy as a main determinant and examined confounding by indication and/or shared familial risk factors.

| **Author, year** | **Study design** | **Total** | **Asthma Outcome** | **Main finding**  risk estimate (95% CI) | **Confounding by indication or shared familial risk factors** |
| --- | --- | --- | --- | --- | --- |
| Stokholm 2014 (Denmark) [1] | Register cohort | 846 689 | 0-13 years, ≥ 1 prescription | aIRR 1.18 (1.15-1.20) | Association was similar before, during and after pregnancy. Association found for all indications, but stronger for antibiotics for RTIs. |
| Stensballe 2013 (Denmark) [2] | Population birth cohort | 30 675 | 0-5 years, ≥ 1 prescription | aHR 1.18 (1.10-1.27) | Risk increased in mothers using antibiotics for non-RTIs compared to other antibiotics. |
| Örtqvist 2014 [3] and 2017 (Sweden) [4] | Register cohort | 493 785 | 0- 5 years, ≥ 2 prescriptions | aHR 1.28 (1.25-1.32) | Association found for all indications, but stronger for antibiotics for RTIs. Similar before, during and after pregnancy. Paternal antibiotic use was associated with asthma in children < 2.5 but not in children ≥ 2.5 years of age. In the sibling analysis associations disappeared. |
| Lapin 2015 (USA) [5] | Prospective high-risk cohort | 298 | 0-3 years, mother-reported | aOR 3.12 (1.44 - 6.77) | Association between prenatal antibiotic use and asthma by 3 years was similar within a subset analysis of mothers with no history of asthma and within a subset of children who did not use antibiotics in the first year. |
| Chu 2015 (USA) [6] | Prospective birth cohort | 39 907 | 0-7 years, based on ICD | aOR 1.21 (1.08–1.36) for  penicillin; aOR 1.72 (1.14–2.59) for chloramphenicol | Association was stronger for the antibiotics in the first trimester of pregnancy. Antibiotic use 4 weeks before pregnancy was not associated with risk of asthma. |
| Loewen 2018 (Canada) [7] | Register cohort | 213 661 | 5-19 years, prescriptions | aHR 1.23 (1.20-1.27) | Association was similar before, during and after pregnancy. |
| Metsälä 2014 (Finland) [8] | Register-based nested case-control study | 6 690 pairs | 3-6 years, reimbursement for asthma drugs | aOR 1.31 (1.21-1.42) | Association found for all indications, but stronger for antibiotics for RTIs. In the sibling analysis association disappeared. |
| Mulder 2016 (Netherlands) [9] | Register based case-sibling and case-control | 2 456 and 26 278 | 0-5 years, ≥ 3 prescriptions | case-sibling: aOR 1.06 (0.85-1.32); 3^rd^ trim: aOR 1.37 (1.02-1.83); case-control: aOR 1.45 (1.33-1.58); 3^rd^ trim 1.40 (1.15-1.47) | Took into account time-trend bias in a case-sibling design and correlation of antibiotic use at different time points. |
| Momen 2021 (Denmark) [10] | Register cohort | 407 804 | from 5 years to the first onset of asthma | aHR 1.21 (1.18–1.24); sibling analysis aHR 0.96 (0.90–1.03) | Association was similar before, during and after pregnancy. In the sibling analysis association disappeared. |
| Yoshida 2018 (Japan) [11] | Retrospective register cohort | 83 470 | 1-6 years, based on ICD and ≥ 2 prescriptions | 1-<3 years: HR 1.18 (1.08-1.30); 3-<6 years: HR 1.09 (0.97-1.22) | In the sibling cohort, association remained significant. |
| Uldbjerg 2021 (Denmark) [12] | Population birth cohort | 32 651 | 11 years, mother reported | aOR 1.13 (1.04-1.23) | Association was stronger for antibiotic exposure  in the second to third trimester. |
| Turi 2021 (USA) [13] | Prospective cohort | 84 214 mother-child pairs | 4.5-6 years, pharmacy claims and asthma-related healthcare encounters | aOR 1.23 (1.18-1.28) | Association stronger when first dose in early pregnancy. Effect modification by maternal asthma, association significant only among those without maternal asthma. |
| da Cunha 2021 (UK) [14] | Birth cohort | 13 685 | 5-8 years, diagnostic code and ≥ 1 prescription | 3rd trimester of pregnancy: aOR 1.40 (1.05-1.87) | There was no association for antibiotic use before pregnancy (0-12months) |

CI, confidence interval; HR, hazard ratio; ICD, International Classification of Diseases; IRR, incidence rate ratio OR, odds ratio; RTI, respiratory tract infections

**Supplementary Fig. S1 Selection of the study population. MoBa, Norwegian Mother, Father and Child Cohort Study**

#

# **Supplementary Table S2. Anatomical therapeutic chemical (ATC) codes used to classify antibiotics.**

|  | **ATC code** | **Description** |
| --- | --- | --- |
| **ANTIBIOTICS** |  |  |
| Penicillin V | J01CE | Beta-lactamase sensitive penicillins |
| Extended spectrum penicillins | J01CA | Penicillins with extended spectrum |
|  | J01CF | Beta-lactamase resistant penicillins |
|  | J01CG | Beta-lactamase inhibitors |
|  | J01CR | Combinations of penicillins |
| Other specified antibiotics | J01A | Tetracyclines |
|  | J01B | Amphenicols |
|  | J01D | Other beta-lactam antibacterials |
|  | J01E | Sulfonamides and trimethoprim |
|  | J01F | Macrolides, lincosamides and streptogramins |
|  | J01G | Aminoglycoside antibacterials |
|  | J01M | Quinolone antibacterials |
|  | J01R | Combinations of antibacterials |
|  | J01X | Other antibacterials |
| Unspecified * | - | - |

^*^ Include antibiotics not designated by name or antibiotic type and therefore could not be grouped according to the ATC classification system.

**Supplementary Fig. S2 Directed acyclic graphs illustrating our hypothetical causal model of A) the association between maternal antibiotic use during pregnancy and offspring asthma at 7 years and B) the association between maternal experience of infections during pregnancy and offspring asthma at 7 years.**

BMI, body mass index; RTIs, respiratory tract infections, UTIs, urinary tract infections

**Supplementary Table S3. Incidence of specific antibiotic use and asthma at 7 years in MoBa**

| **Maternal antibiotic use** | **N (%)** | **N (%) Asthma** |
| --- | --- | --- |
| **Total** | 53417 (100.0) | 2201 (4.1) |
| **Any antibiotic** | 8323 (15.6) | 419 (5.0) |
| **Penicillin V** |  |  |
| No | 50994 (95.5) | 2074 (4.1) |
| Yes | 2423 (4.5) | 127 (5.2) |
| **Extended spectrum penicillins** |  |  |
| No | 50288 (94.1) | 2029 (4.0) |
| Yes | 3129 (5.9) | 172 (5.5) |
| **Macrolides** |  |  |
| No | 52459 (98.2) | 2142 (4.1) |
| Yes | 958 (1.8) | 59 (6.2) |
| **Other specified systemic antibiotics** |  |  |
| No | 52197 (97.7) | 2138 (4.1) |
| Yes | 1220 (2.3) | 63 (5.2) |
| **Unspecified antibiotics** |  |  |
| No | 51633 (96.7) | 2113 (4.1) |
| Yes | 1784 (3.3) | 88 (4.9) |

**Supplementary Table S4. The association between maternal antibiotic use during pregnancy and the risk of asthma at 7 years in MoBa**

| **Antibiotic use** | **N** | **% Asthma** | **Unadjusted RR**  **(95% CI)** | **Adjusted RR for common covariates (95% CI)^a^** | **Adjusted RR + infections (95% CI)^b^** | **Adjusted RR + mediators (95% CI)^c^** |
| --- | --- | --- | --- | --- | --- | --- |
| **Total** | **53 417** | 4.1 |  |  |  |  |
| No use | 45 094 | 4.0 | 1 | 1 | 1 | 1 |
| Yes (≥ 1) | 8323 | 5.0 | 1.27 (1.15 - 1.41) | 1.23 (1.11 - 1.37) | 1.15 (1.02 - 1.30) | 1.11 (0.98 - 1.26) |
|  |  |  |  |  |  |  |
| 1 | 6454 | 4.6 | 1.16 (1.03 - 1.31) | 1.11 (0.98 - 1.26) | 1.05 (0.92 - 1.21) | 1.02 (0.89 - 1.16) |
| 2 or more | 1869 | 6.6 | 1.67 (1.40 - 1.99) | 1.66 (1.39 - 1.98) | 1.54 (1.27 - 1.88) | 1.50 (1.24 - 1.82) |
|  |  |  |  |  |  |  |
| **Timing of pregnancy^d^** |  |  |  |  |  |  |
| Reference | 52 649 | 4.1 | 1 | 1 | 1 | 1 |
| Only early (< 17 weeks) | 396 | 5.8 | 1.42 (0.95 - 2.12) | 1.46 (0.99 - 2.16) | 1.30 (0.88 - 1.94) | 1.28 (0.87 - 1.90) |
| Only late (>=17 weeks) | 310 | 7.7 | 1.90 (1.29 - 2.79) | 1.83 (1.25 - 2.69) | 1.65 (1.12 - 2.43) | 1.57 (1.07 - 2.32) |
| Both periods of pregnancy | 62 | 6.5 | 1.58 (0.61 - 4.08) | 1.60 (0.62 - 4.12) | 1.37 (0.53 - 3.56) | 1.46 (0.57 - 3.73) |
|  |  |  |  |  |  |  |
| **Outside of pregnancy^d^** |  |  |  |  |  |  |
| No use | 50 979 | 4.0 | 1 | 1 | 1 | 1 |
| Only during pregnancy | 1769 | 6.4 | 1.60 (1.33 - 1.92) | 1.60 (1.33 - 1.92) | 1.48 (1.22 - 1.80) | 1.47 (1.21 - 1.78) |
| Only after pregnancy | 569 | 3.5 | 0.87 (0.56 - 1.34) | 0.89 (0.58 - 1.36) | 0.88 (0.57 - 1.35) | 0.84 (0.55 - 1.27) |
| Both | 100 | 9.0 | 2.23 (1.19 - 4.16) | 2.28 (1.24 - 4.19) | 2.07 (1.12 - 3.84) | 1.80 (0.97 - 3.36) |

^a^ Adjusted for maternal age, parity, pre-pregnancy BMI, asthma, smoking during pregnancy and education (confounders); ^b^ in addition adjusted for

maternal infections during pregnancy (RTI and UTI) (confounders); ^c^ in addition, adjusted for child’s sex, preterm birth, caesarean section, birth

weight and breastfeeding by 18 months, and child antibiotic use and respiratory tract infections by 18 months (mediators); ^d^ No antibiotic use or use

only for one infection was compared to use of antibiotics for two or more infections

**Supplementary Table S5.** **The association between maternal infections during pregnancy and the risk of asthma at 7 years (MoBa cohort)**

| **Infections** | **N** | **% Asthma** | **Unadjusted RR**  **(95% CI)** | **Adjusted RR for common covariates (95% CI)^a^** | **Adjusted RR + antibiotics (95% CI)^b^** | **Adjusted RR + mediators (95% CI)^c^** |
| --- | --- | --- | --- | --- | --- | --- |
| **All RTIs** |  |  |  |  |  |  |
| No | 20 747 | 4.0 | 1 | 1 | 1 | 1 |
| Yes (≥1) | 32 670 | 4.2 | 1.07 (0.98 - 1.17) | 1.07 (0.98 - 1.17) | 1.05 (0.96 - 1.15) | 1.02 (0.93 - 1.11) |
|  |  |  |  |  |  |  |
| One | 18 491 | 3.9 | 0.98 (0.89 - 1.08) | 0.99 (0.90 - 1.10) | 0.98 (0.89 - 1.09) | 0.96 (0.87 - 1.06) |
| Two or more | 14 179 | 4.7 | 1.19 (1.07 - 1.31) | 1.18 (1.07 - 1.31) | 1.14 (1.03 - 1.27) | 1.09 (0.98 - 1.21) |
|  |  |  |  |  |  |  |
| **LRTIs** |  |  |  |  |  |  |
| No | 51 685 | 4.1 | 1 | 1 | 1 | 1 |
| Yes (≥ 1) | 1 732 | 5.8 | 1.44 (1.18 - 1.74) | 1.30 (1.07 - 1.57) | 1.18 (0.96 - 1.44) | 1.11 (0.91 - 1.36) |
|  |  |  |  |  |  |  |
| **URTIs** |  |  |  |  |  |  |
| No | 38 408 | 3.9 | 1 | 1 | 1 | 1 |
| Yes^d^ | 15 009 | 4.7 | 1.19 (1.09 - 1.30) | 1.19 (1.09 - 1.30) | 1.15 (1.05 - 1.26) | 1.11 (1.02 - 1.22) |
|  |  |  |  |  |  |  |
| Common colds or Influensa |  |  |  |  |  |  |
| 0-1 | 43 408 | 4.0 | 1 | 1 |  | 1 |
| Two or more | 10 009 | 4.6 | 1.16 (1.05 - 1.28) | 1.14 (1.03 - 1.27) | 1.13 (1.02 - 1.25) | 1.10 (1.00 - 1.22) |
| Throat or ear infections |  |  |  |  |  |  |
| No | 45 794 | 4.0 | 1 | 1 | 1 | 1 |
| Yes (≥ 1) | 7 623 | 5.0 | 1.26 (1.13 - 1.40) | 1.26 (1.13 - 1.41) | 1.20 (1.06 - 1.34) | 1.15 (1.03 - 1.30) |
|  |  |  |  |  |  |  |
| **Urinary tract infections** |  |  |  |  |  |  |
| No | 47 894 | 4.0 | 1 | 1 | 1 | 1 |
| Yes (≥1) | 5 523 | 5.1 | 1.28 (1.13 - 1.45) | 1.26 (1.11 - 1.42) | 1.15 (1.00 - 1.32) | 1.16 (1.01 - 1.33) |

^a^ Adjusted for maternal age, parity, pre-pregnancy BMI, asthma, smoking during pregnancy and education (confounders); ^b^ in addition adjusted for

maternal antibiotic use; ^c^ in addition, adjusted for child’s sex, preterm birth, caesarean section, birth weight and breastfeeding by 18 months and child

antibiotic use and respiratory tract infections by 18 months (mediators) ^d^ Having URTIs included two or more common colds or influenza or any throat or

ear infections

**Supplementary Table S6. The association between maternal antibiotic use during and outside pregnancy and asthma at 7 years in the register-based cohort 2004-2011 (N=417,548)**

| **Antibiotic use during pregnancy** | **N** | **% Asthma** | **Unadjusted RR (95% CI)** | **Adjusted RR**  **(95% CI)^a^** | **Adjusted RR**  **(95% CI)^b^** |
| --- | --- | --- | --- | --- | --- |
| **Total** | 417 548 | 3.8 |  |  |  |
| No use | 299 261 | 3.5 | 1 | 1 | 1 |
| Yes (≥ 1) | 118 287 | 4.4 | 1.28 (1.24 - 1.32) | 1.21 (1.16 - 1.25) | 1.16 (1.12 - 1.21) |
|  |  |  |  |  |  |
| One course | 43 603 | 4.4 | 1.26 (1.20 - 1.32) | 1.20 (1.14 - 1.27) | 1.17 (1.11 - 1.23) |
| Two or more courses | 74 684 | 4.5 | 1.29 (1.24 - 1.34) | 1.21 (1.16 - 1.26) | 1.16 (1.11 - 1.21) |
|  |  |  |  |  |  |
| Only early (< 17 weeks) pregnancy | 34 695 | 4.4 | 1.26 (1.19 - 1.33) | 1.18 (1.12 - 1.25) | 1.13 (1.07 - 1.20) |
| Only late (>=17 weeks) pregnancy | 65 529 | 4.3 | 1.24 (1.19 - 1.29) | 1.19 (1.14 - 1.24) | 1.16 (1.10 - 1.21) |
| Both periods of pregnancy | 18 063 | 5.0 | 1.45 (1.36 - 1.55) | 1.32 (1.22 - 1.42) | 1.25 (1.16 - 1.34) |
|  |  |  |  |  |  |
| **Outside of pregnancy (0 vs. ≥ 1)** |  |  |  |  |  |
| No use | 202 324 | 3.2 | 1 | 1 | 1 |
| only 6 months before pregnancy | 25 734 | 3.9 | 1.23 (1.15 - 1.31) | 1.16 (1.08 - 1.24) | 1.12 (1.04 - 1.20) |
| only during pregnancy | 64 263 | 4.1 | 1.27 (1.21 - 1.33) | 1.19 (1.14 - 1.25) | 1.16 (1.11 - 1.22) |
| only 6 months after pregnancy | 43 580 | 4.0 | 1.24 (1.18 - 1.31) | 1.23 (1.16 - 1.30) | 1.18 (1.12 - 1.25) |
| missing | 81 647 | 4.7 |  |  |  |

**^a^** Adjusted for cohort year, maternal age, parity, smoking and asthma during pregnancy and education; ^b^ In addition adjusted for child’s sex, preterm

birth, caesarean section, birth weight and antibiotic use by 18 months

**Supplementary Table S7. The association between maternal antibiotic use during and outside pregnancy and asthma at 13 years in the register-based cohort 2004-2005 (N=67 098)**

| **Maternal antibiotic use during pregnancy** | **N** | **% Asthma** | **Unadjusted**  **(95% CI)** | **Adjusted RR**  **(95% CI)^a^** | **Adjusted RR**  **(95% CI)^b^** |
| --- | --- | --- | --- | --- | --- |
| Total | 67 098 | 4.4 |  |  |  |
| No use | 48 485 | 4.2 | 1 |  |  |
| Yes (≥ 1) | 18 613 | 4.9 | 1.17 (1.08-1.26) | 1.13 (1.03-1.23) | 1.09 (1.00-1.19) |
|  |  |  |  |  |  |
| One course | 7 499 | 4.8 | 1.14 (1.02 - 1.28) | 1.10 (0.97 - 1.24) | 1.06 (0.94 - 1.20) |
| Two or more courses | 11 114 | 4.9 | 1.19 (1.08 - 1.30) | 1.15 (1.03 - 1.27) | 1.11 (1.00 - 1.24) |
|  |  |  |  |  |  |
| Only early (< 17 weeks) pregnancy | 5 610 | 4.7 | 1.14 (1.02 - 1.28) | 1.10 (0.96 - 1.27) | 1.07 (0.93 - 1.23) |
| Only late (>=17 weeks) pregnancy | 10 272 | 4.9 | 1.18 (1.05 - 1.32) | 1.15 (1.03 - 1.28) | 1.11 (1.00 - 1.24) |
| Both periods of pregnancy | 2 731 | 5.0 | 1.20 (1.05 - 1.38) | 1.10 (0.90 - 1.33) | 1.05 (0.87 - 1.28) |
|  |  |  |  |  |  |
| **Outside of pregnancy (0 vs. ≥ 1)** |  |  |  |  |  |
| No use | 21 572 | 3.9 | 1 | 1 | 1 |
| only 6 months before pregnancy | 2 537 | 4.1 | 1.05 (0.86 - 1.29) | 1. 00 (0.80 - 1.26) | 0.98 (0.78 - 1.22) |
| only during pregnancy | 6 846 | 4.3 | 1.12 (0.99 - 1.28) | 1.06 (0.92 - 1.23) | 1.04 (0.90 - 1.21) |
| only 6 months after pregnancy | 4 514 | 4.2 | 1.08 (0.93 - 1.26) | 1.12 (0.95 - 1.33) | 1.10 (0.93 - 1.31) |
| missing | 31 629 | 4.8 |  |  |  |

**^a^** Adjusted for cohort year, maternal age, parity, smoking and asthma during pregnancy and education; ^b^ In addition adjusted for child’s sex, preterm birth, caesarean section, birth weight and antibiotic use by 18 months

**Supplementary Table S8. Risk of asthma at 7 years according to antibiotic use in the register-based cohort, using a sibling-matched design**

| **Antibiotic use during pregnancy** | **Unadjusted (95% CI)** | **Adjusted RR (95% CI)^a^** | **Adjusted RR (95% CI)^b^** |
| --- | --- | --- | --- |
|  | N=13 498 | N=9326 | N=9326 |
| No |  |  |  |
| Yes (≥ 1) | 0.95 (0.88 - 1.03) | 0.95 (0.82 - 1.03) | 0.96 (0.88-1.04) |
|  |  |  |  |
| One course | 0.94 (0.84-1.04) | 0.92 (0.82-1.03) | 0.93 (0.83-1.04) |
| Two or more courses | 0.97 (0.88-1.07) | 0.97 (0.88-1.08) | 0.98 (0.88-1.09) |

^a^ Adjusted for maternal age, parity, asthma, smoking during pregnancy and education; ^b^ In addition adjusted for child sex, preterm birth, caesarean section, birth weight and antibiotic use by 18 months; The number of included sibling sets was 6 314 in the unadjusted model and 4 412 in the adjusted model. Altogether 6 392 (47.3%) and 4 458 (47.8%) of children included in the model had asthma and 5 567 and 3 852 of the children had discordant exposures, respectively.

**Supplementary Table S9. The association of maternal infections and the use of antibiotics during pregnancy with asthma at 7 in MoBa cohort (mutually exclusive indication categories)**

| **Infections and antibiotic use for that indication during pregnancy** | **N** | **Asthma %** | **Unadjusted (95% CI)** | **Adjusted RR**  **(95% CI)^a^** | **Adjusted RR**  **(95% CI)^b^** |
| --- | --- | --- | --- | --- | --- |
| **Total** | 53 417 | 4.1 |  |  |  |
| **All RTIs** |  |  |  |  |  |
| No RTI + no antibiotic use | 18 822 | 3.8 | 1 |  |  |
| RTI + no antibiotic use for that indication | 29 981 | 4.1 | 1.07 (0.98 - 1.17) | 1.07 (0.98 - 1.17) | 1.03 (0.94 - 1.13) |
| RTI + antibiotic use for that indication | 2 689 | 5.5 | 1.42 (1.20 - 1.69) | 1.39 (1.17 - 1.66) | 1.28 (1.07 - 1.52) |
| missing | 1 925 | 4.9 |  |  |  |
|  |  |  |  |  |  |
| **LRTIs** |  |  |  |  |  |
| No LRTI + no antibiotic use | 44 517 | 3.9 | 1 |  |  |
| LRTI + no antibiotic for that indication | 984 | 6.1 | 1.56 (1.21 - 2.00) | 1.40 (1.09 - 1.79) | 1.30 (1.02 - 1.66) |
| LRTI + antibiotic for that indication | 748 | 5.5 | 1.40 (1.03 - 1.89) | 1.26 (0.93 - 1.71) | 1.17 (0.86 - 1.58) |
| missing | 7 168 | 5.0 |  |  |  |
|  |  |  |  |  |  |
| **URTIs** |  |  |  |  |  |
| No URTI + no antibiotic use | 18 908 | 3.9 | 1 |  |  |
| URTI + no antibiotic for that indication | 30 241 | 4.1 | 1.07 (0.97 - 1.17) | 1.06 (0.97 - 1.17) | 1.03 (0.94 - 1.13) |
| URTI + antibiotic for that indication | 2 096 | 5.5 | 1.43 (1.18 - 1.73) | 1.43 (1.18 - 1.74) | 1.32 (1.09 - 1.60) |
| missing | 2 172 | 5.0 |  |  |  |
|  |  |  |  |  |  |
| **UTIs** |  |  |  |  |  |
| No UTI + no antibiotic use | 43 270 | 3.9 | 1 |  |  |
| UTI + no antibiotic use for that indication | 2 552 | 5.1 | 1.30 (1.10 - 1.55) | 1.27 (1.06 - 1.51) | 1.23 (1.03 - 1.46) |
| UTI + antibiotic use for that indication | 2 971 | 5.1 | 1.32 (1.12 - 1.55) | 1.29 (1.10 - 1.52) | 1.27 (1.08 - 1.50) |
| missing | 4 624 | 4.9 |  |  |  |

^a^ Adjusted for maternal age, parity, pre-pregnancy BMI, asthma, smoking during pregnancy and education (confounders); ^b^ In addition adjusted for and child’s sex, preterm birth, birth weight, antibiotic use and respiratory tract infections by 18 months

**Supplementary Table S10. Indication categories of antibiotic prescription during pregnancy and asthma at 7 (N=417,548) and 13 years (N=67 098) in the register-based cohort**

|  | **Asthma at 7 years** | | | | **Asthma at 13 years** | | | |
| --- | --- | --- | --- | --- | --- | --- | --- | --- |
| **Categorized antibiotics based on indication** | **N** | **Unadjusted RR (95% CI)** | **Addjusted RR (95% CI)^a^** | **Adjusted RR**  **(95% CI)^b^** | **N** | **Unadjusted RR (95% CI)** | **Adjusted RR (95% CI)^a^** | **Adjusted RR (95% CI)^b^** |
| **Respiratory tract infections^c^** |  |  |  |  |  |  |  |  |
| No | 355 908 | 1 | 1 | 1 | 56 846 | 1 | 1 | 1 |
| Yes (≥ 1) | 61 640 | 1.27 (1.22 - 1.33) | 1.18 (1.13 - 1.24) | 1.14 (1.09 - 1.19) | 10 252 | 1.16 (1.06 - 1.28) | 1.14 (1.02 - 1.26) | 1.10 (0.99 - 1.22) |
| **Urinary tract infections^d^** |  |  |  |  |  |  |  |  |
| No | 352 202 | 1 | 1 | 1 | 57 353 | 1 | 1 | 1 |
| Yes (≥ 1) | 65 346 | 1.21 (1.17 - 1.26) | 1.16 (1.11 - 1.22) | 1.13 (1.08 - 1.18) | 9 745 | 1.12 (1.02 - 1.24) | 1.05 (0.94 - 1.17) | 1.02 (0.91 - 1.14) |

^a^ Adjusted for cohort year, maternal age, parity, smoking, asthma and education (confounders); ^b^ In addition adjusted for child’s sex, gestational age, birth weight and antibiotic

use by 18 months (mediators); ^c^ trimetroprim (J01E), nitrofurantoin (J01XE01), pivmecillinam (J01CA08), ciprofloksacin (J01MA) and metenamin (J01XX);

^d^ amoxicillin (J01CA04, J01CR02), penicillin V (J01CE) and macrolides (J01FA)

**References**

1. Stokholm J, Sevelsted A, Bønnelykke K, Bisgaard H. Maternal propensity for infections and risk of childhood asthma: a registry-based cohort study. Lancet Respir Med. 2014;8:631.
2. Stensballe LG, Simonsen J, Jensen SM, Bønnelykke K, Bisgaard H. Use of antibiotics during pregnancy increases the risk of asthma in early childhood. J Pediatr. 2013;162:832–8.
3. Örtqvist AK, Lundholm C, Kieler H et al. Antibiotics in fetal and early life and subsequent childhood asthma: nationwide population based study with siblings analysis. BMJ. 2014;349:g6979.
4. Örtqvist AK, Lundholm C, Fang F, Fall T, Almqvist C. Parental antibiotics and childhood asthma—a population-based study. J Allergy Clin Immunol Pract. 2017;5:1451.
5. Lapin B, Piorkowski J, Ownby D et al. The Relationship between Prenatal Antibiotic Use and Asthma in At-Risk Children. Ann Allergy Asthma Immunol. 2015; 114(3):203–7.
6. Chu S, Yu H, Chen Y, Chen Q, Wang B, Zhang J. Periconceptional and Gestational Exposure to Antibiotics and Childhood Asthma. PLoS ONE. 2015;10(10):e0140443.
7. Loewen K, Monchka B, Mahmud SM, Jong G, Azad MB. Prenatal antibiotic exposure and childhood asthma: a population-based study. Eur Respir J. 2018;52:1702070.
8. Metsälä J, Lundqvist A, Virta LJ, Kaila M, Gissler M, Virtanen SM. Prenatal and post-natal exposure to antibiotics and risk of asthma in childhood. Clin Exp Allergy. 2015;45:137–45.
9. Mulder B, Pouwels KB, Schuiling-Veninga CC et al. Antibiotic use during pregnancy and asthma in preschool children: the influence of confounding. Clin Exp Allergy. 2016;46:1214
10. Momen NC, Liu X. Maternal antibiotic use during pregnancy and asthma in children: population-based cohort study and sibling design. Eur Respir J. 2021;57:2000937.
11. Yoshida S, Ide K, Takeuchi M, Kawakami K. Prenatal and early-life antibiotic use and risk of childhood asthma: A retrospective cohort study. Pediatr Allergy Immunol. 2018;29:490.
12. Uldbjerg CS, Miller JE, Burgner D, Pedersen LH, Bech MH. Antibiotic exposure during pregnancy and childhood asthma: a national birth cohort study investigating timing of exposure and mode of delivery. Arch Dis Child. 2021;106:888.
13. Turi KN, Gebretsadik T, Ding T et al. Dose, timing, and spectrum of prenatal antibiotic exposure and risk of childhood asthma. Clin Inf Dis. 2021;72:455.
14. da Cunha SS, Santorelli G, Pearce N et al. Evidence for causal associations between prenatal and postnatal antibiotic exposure and asthma in children, England. Clin Exp Allergy. 2021;51:1438.
